# The impact of underwater waves on ship manoeuvrability: a case study in a fjord

**DOI:** 10.1038/s41598-025-90132-x

**Published:** 2025-02-15

**Authors:** Sandy Grégorio, Daniel Bourgault, Peter S. Galbraith, Cédric Chavanne, Louis Hupé, Alain Richard, Étienne Landry

**Affiliations:** 1 Institut des sciences de la mer, Université du Québec à Rimouski, Rimouski, QC Canada; 2 Fisheries and Oceans Canada, Québec Region, Maurice Lamontagne Institute, Mont-Joli, QC Canada; 3 AREN – Expert Nautique, Rimouski, QC Canada; 4 Laurentian Pilotage Authority, Montréal, QC Canada

**Keywords:** Physical oceanography, Fluid dynamics, Computational science

## Abstract

In 2019, the Motor Vessel *Jaeger Arrow* collided with the Grande-Anse Terminal wharf (Saguenay Fjord, Canada) during docking from unknown causes. However, the timeline of the incident and the ship’s behavior during docking suggest that underwater waves may have caused the collision. Data collected in 2023 using a camera and thermometers confirmed that this area of the fjord regularly experiences underwater waves with wavelengths ranging from 50 to $100~\textrm{m}$, wave heights of 1 and $3~\textrm{m}$, and periods of around $2~\textrm{min}$. These waves frequently collide with and reflect off the wharf, generating currents of 0.1 to $0.3~\textrm{m}\,\textrm{s}^{-1}$. Numerical simulations further illustrate the interactions between the waves and the wharf, highlighting regions near the wharf where wave-induced currents, both inshore and offshore, occur, including areas with near-zero currents that could create a false sense of calm conditions. Importantly, our observations also revealed that large ships, such as the *Jaeger Arrow*, can generate their own underwater waves, potentially compromising docking operations. While we cannot definitively confirm that underwater waves caused the incident involving the *Jaeger Arrow*, our study offers a plausible explanation: the ship may have been caught in a wavetrain reflecting off the wharf, leading to unpredictable movement during docking. These results highlight the potential risks posed by underwater waves to ship safety and maneuverability during docking operations, a topic under-explored in existing scientific literature.

## Introduction

Underwater waves, also called internal waves by physical oceanographers, are physically analogous to the surface waves that make boats rock and roll except that, instead of propagating at the density interface that separates the light atmosphere from the dense ocean, they propagate at depth between the density layers that characterize the vertical structure of the ocean. Although difficult to detect without specialized instruments and expertise, underwater waves are widespread in the ocean, including coastal seas, gulfs, estuaries and fjords^1^. Technically speaking, the underwater waves that interests us here are called internal solitary waves or nonlinear internal waves^1^ but hereafter, the terms waves, underwater waves, internal waves, internal solitary waves, and nonlinear internal waves will be treated as synonymous in the context of this study, unless otherwise specified.

**Fig. 1:**
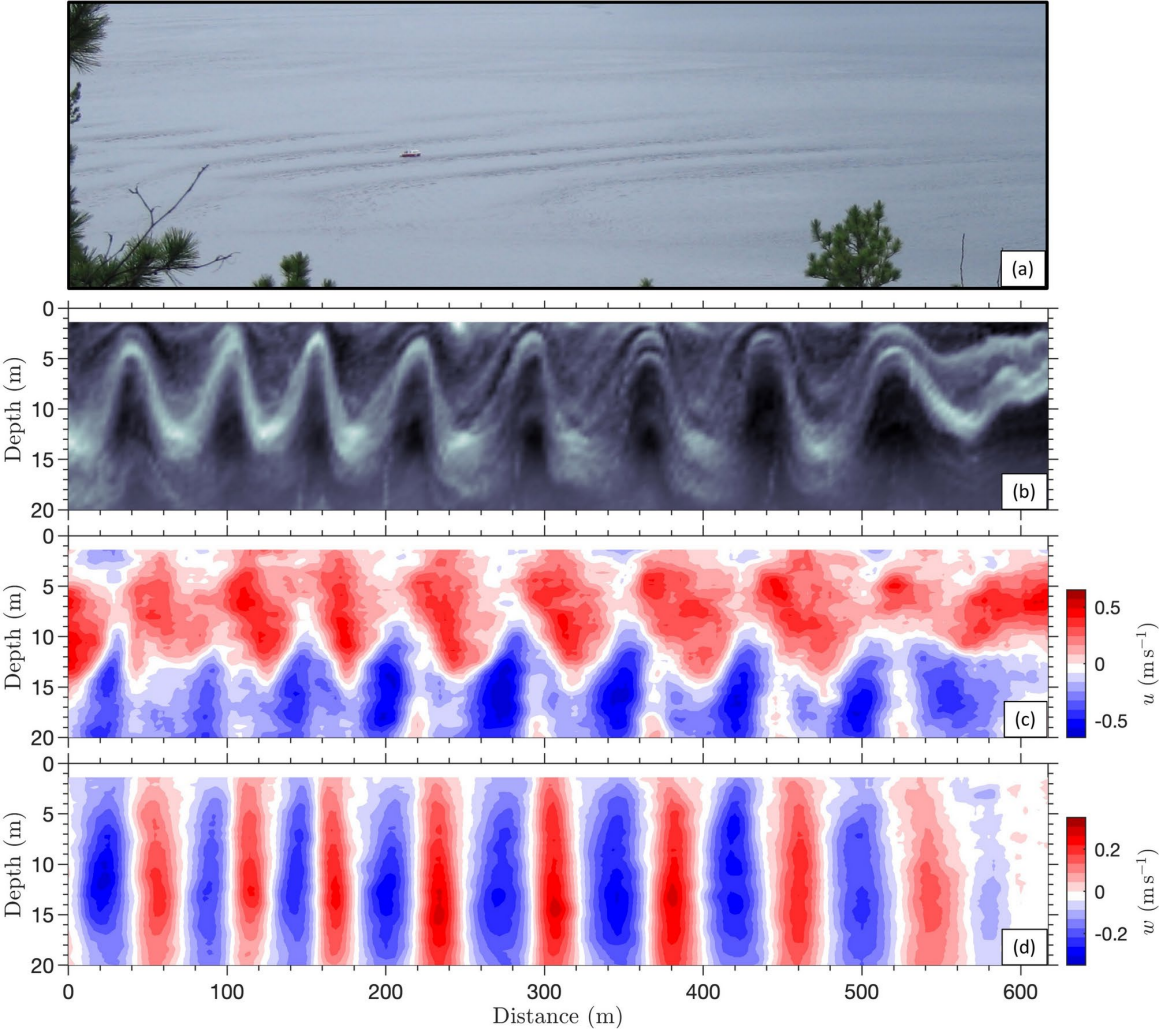
Example of a rightward-propagating large-amplitude naturally-occurring underwater wavetrain observed in the Saguenay Fjord near Anse-de-Roche, located roughly 80 km downstream from Grande-Anse, on 9 July 2007 (after Janes (2008)^2^). (**a**) Photo showing the sea-surface signature of the underwater wavetrain. The 8-m long boat seen in the image is the *Krill*, the Department of Fisheries and Oceans Canada research boat used for sampling these waves. (**b**) Echogram showing the corresponding internal structure of the underwater wavetrain; and (**c**) the corresponding horizontal currents (positive towards the right); and (**d**) the vertical currents (positive upwards), measured from the *Krill* with a 600 kHz Acoustic Doppler Current Profiler (ADCP). Note that the horizontal axis has been corrected for the Doppler shift caused by sampling a propagating wavetrain.

**Fig. 2:**
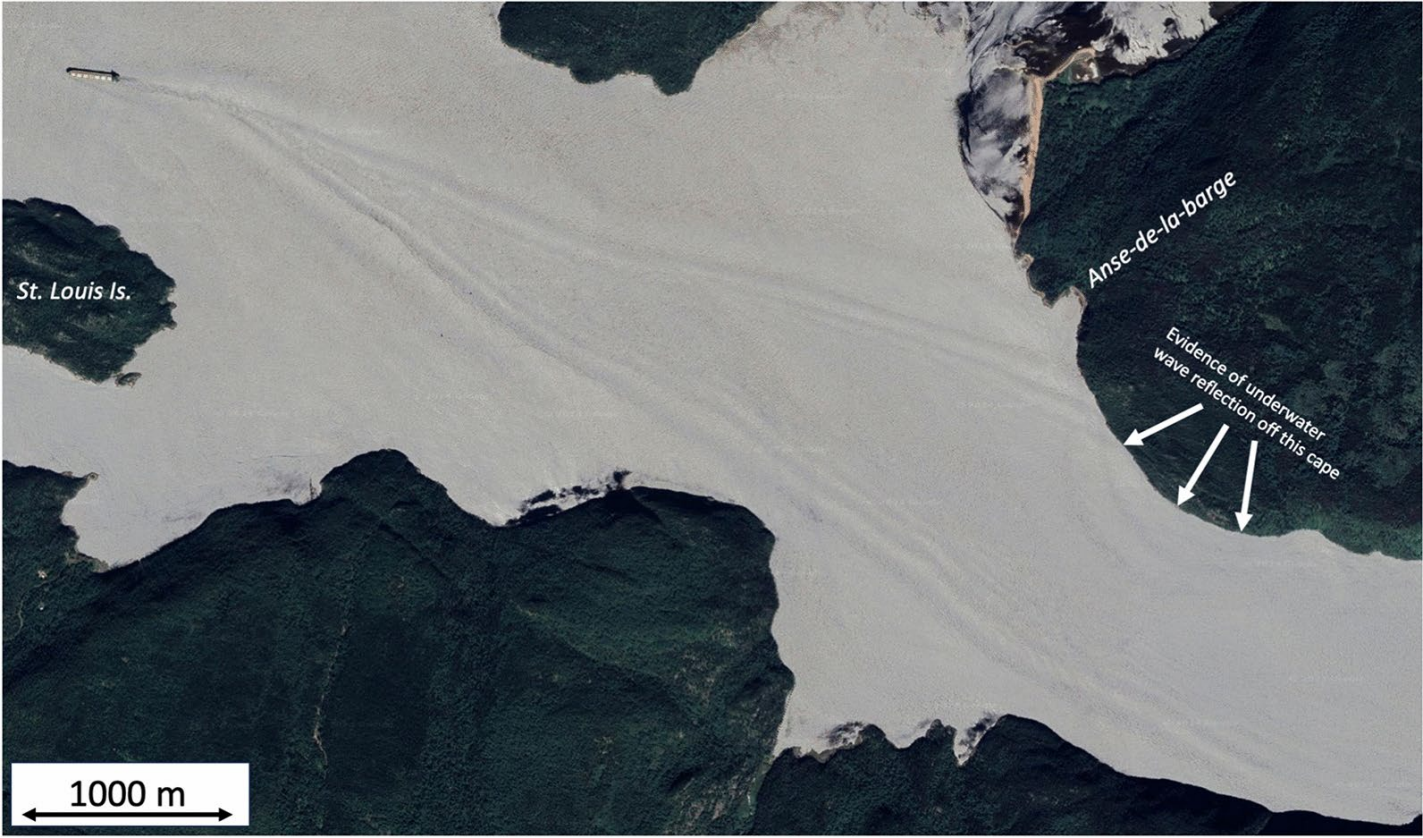
Example of the sea-surface signature of underwater waves generated by a vessel sailing in the Saguenay Fjord near St. Louis Island, seen as the V pattern behind the ship, as observed in other fjords^3,4^. The distance between the bands forming the V pattern is of the order of $100~\textrm{m}$, consistent with the expected dimension of underwater waves in this fjord. Evidence of underwater wave reflection are seen around the cape near Anse-de-la-barge. The satellite image was extracted from Google Earth at $48^{\circ } 15.0358\mathrm {'} \text {N}$, $69^{\circ } 59.0649\mathrm {'} \text {W}$, $4 \text {m}$. Map data (2021-07-03): Google, CNES / Airbus, Maxar Technologies.

While surface waves are mainly caused by the wind, underwater waves may be naturally generated by several different mechanisms that are not all well understood. For example, they can be generated by the flow perturbation caused by bathymetric obstacles^1,5,6^, by the collision of two water masses of different densities^7–9^ or by the evolution and transformation of much longer period internal waves such as internal tides or internal seiches^1,10,11^, to name a few mechanisms. Underwater waves can also be anthropogenically generated. Indeed, similar to how ships generate surface waves while underway, underwater waves can also be generated by steaming ships, especially in highly-stratified fjords where the pycnocline lies within ships draughts^3,4,12–14^. The phenomenon has been coined *dead-water*^15^ because the process tends to slow down ships due to the additional wave drag so-induced.

In coastal waters, underwater waves are generally much larger and longer than surface waves. Depending on the background conditions of the ocean in which they evolve, they may be characterized with heights of several meters, as for example in estuaries and fjords^8,16,17^ (Fig. 1), to hundreds of meters, as for example in the South China Sea^18^. Although underwater waves can be huge, they go largely unnoticed to mariners as they induce very little, often imperceptible, modification of the sea surface height and roughness. When they are perceptible, generally during calm conditions, they manifest themselves at the surface as alternating banded stripes of smooth and rippled water (Figs. 1a and 2). They induce large horizontal and vertical current variations throughout a significant portion of the water depth, up to roughly $\pm 2~\mathrm {m/s}$ ($\sim 4$ knots) in the horizontal and $\pm 0.5~\mathrm {m/s}$ ($\sim 1$ knot) in the vertical, over only a few minutes^8,19,20^. As such, underwater waves may affect the manoeuverability, stability and safety of vessels, drillships and oil rigs^21^. There are even some indications that underwater waves may have been responsible for the sinking of two American nuclear submarines in the 1960’s^22^, as well as an Indonesian submarine in April 2021^23^ by having uncontrollably deported them vertically below their safety depth. Another accident of a Russian submarine that occurred in the Strait of Gibraltar was hypothesized to have been caused by underwater waves that carried the submarine upward before it collided with a surface ship^24^.

However, those are mostly speculative as there is little direct scientific evidence to demonstrate that underwater waves may have caused any significant incident on ships, floating marine infrastructures or submarines. One exception is from Ghomsi (2015)^25^ who attributed the breaking of a mooring hawser of a Floating Storage Offloading (FSO) in the Gulf of Guinea to the passage of a particularly strong underwater wave. Some authors^26–29^ have motivated their study on underwater waves by referring to a study by Fraser (1999)^30^ who apparently reported and documented several cases of “costly and dangerous incidents”^28^ caused by underwater waves running into oil rigs in Southeast Asia. However, we have not been able to obtain a copy of that reference, either online, from the authors who have cited it – whom we contacted directly – or from our university librarian, who conducted an unsuccessful search. For all practical purposes, this reference is unavailable and should no longer be cited. But absence of evidence is not evidence of absence. Given the size and energy that underwater waves carry, it is not unreasonable to think that, under some circumstances, these waves may impact marine operations as previously hypothesized^21^.

Here, we report on an intriguing maritime incident that involved the collision of a ship with a wharf during a routine docking in calm weather conditions in the Saguenay Fjord (Canada). The report of the incident and its chronology were provided by the Laurentian Pilotage Authority (LPA), the Canadian Crown Corporation responsible for administering marine pilotage in the St. Lawrence River and Saguenay Fjord. Our analysis of the incident based on the ship behaviour, on new field observations, on theory and on idealized numerical simulations suggest that the incident was caused by underwater waves. While the analysis of this study does not prove beyond any doubt that the incident was caused by underwater waves, it brings new information on a potentially important subject for ship safety for which the scientific literature is almost inexistent.

## The event

**Fig. 3:**
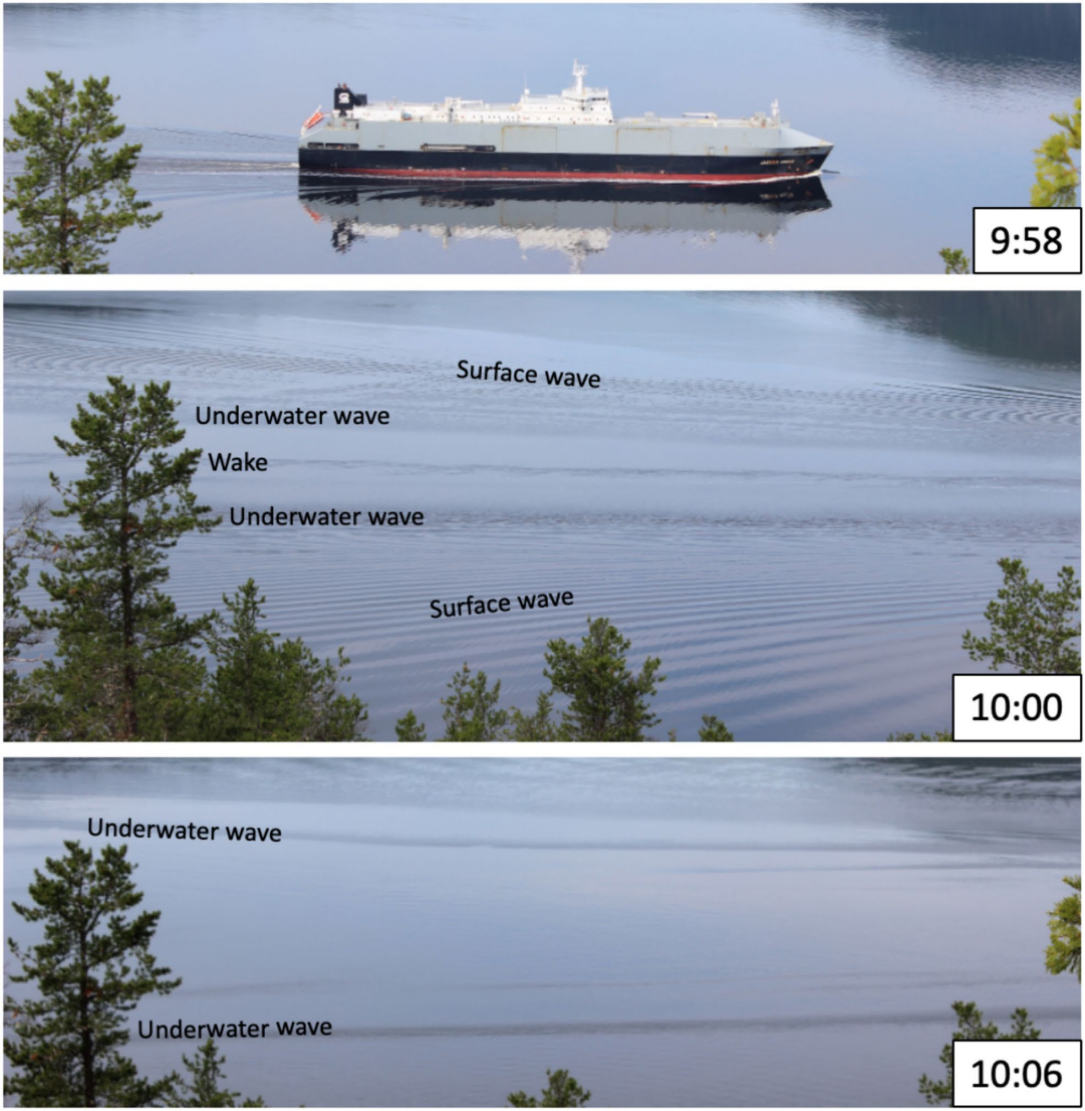
(top) The MV *Jaeger Arrow* photographed while sailing in the Saguenay Fjord near Anse-de-Roche in July 2018. (middle) The sea surface perturbations she induced 2 minutes later which consist of her turbulent wake, surface waves and underwater waves. (bottom) Six minutes later, the turbulent wake has dissipated, the surface waves have propagated away while the underwater waves are still present and slowly propagating. These unpublished photos were coincidentally captured a year prior to the incident described in this paper during an unrelated field experiment carried out by some of us (Bourgault, Galbraith and Chavanne). Photo credit: Jérôme Lemelin.

**Fig. 4:**
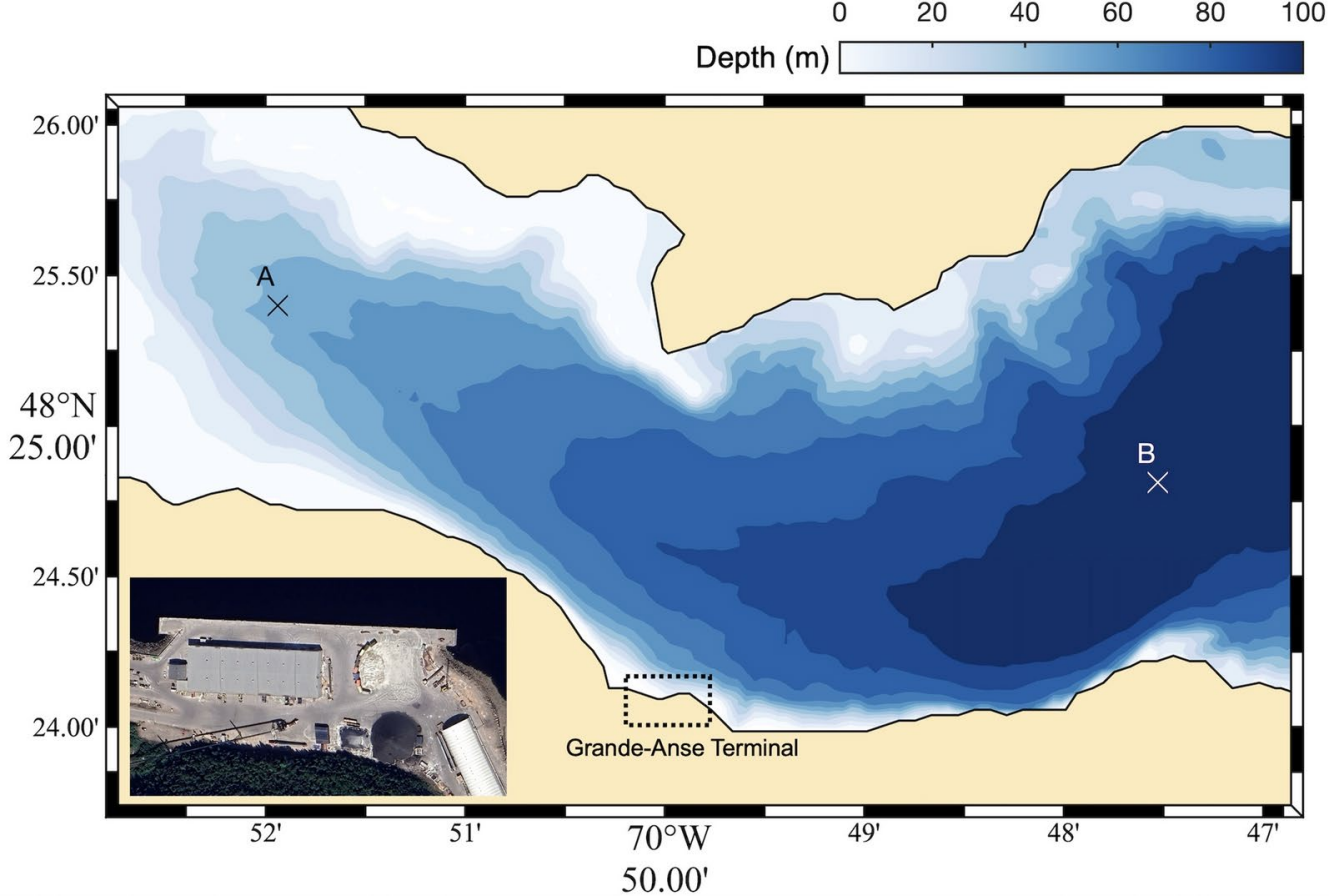
Map of a segment of the Upper Saguenay Fjord centered around the Grande-Anse Terminal of Port Saguenay (dashed box). The crosses indicate the positions where the density measurements presented in Fig. 6 have been collected. The inset shows a satellite image of the terminal extracted from Google Earth at $48^{\circ } 24.0607\mathrm {'} \text {N}$, $70^{\circ } 49.939\mathrm {'} \text {W}$, $10 \text {m}$. Map data (2023-06-03): Google, Airbus.

**Fig. 5:**
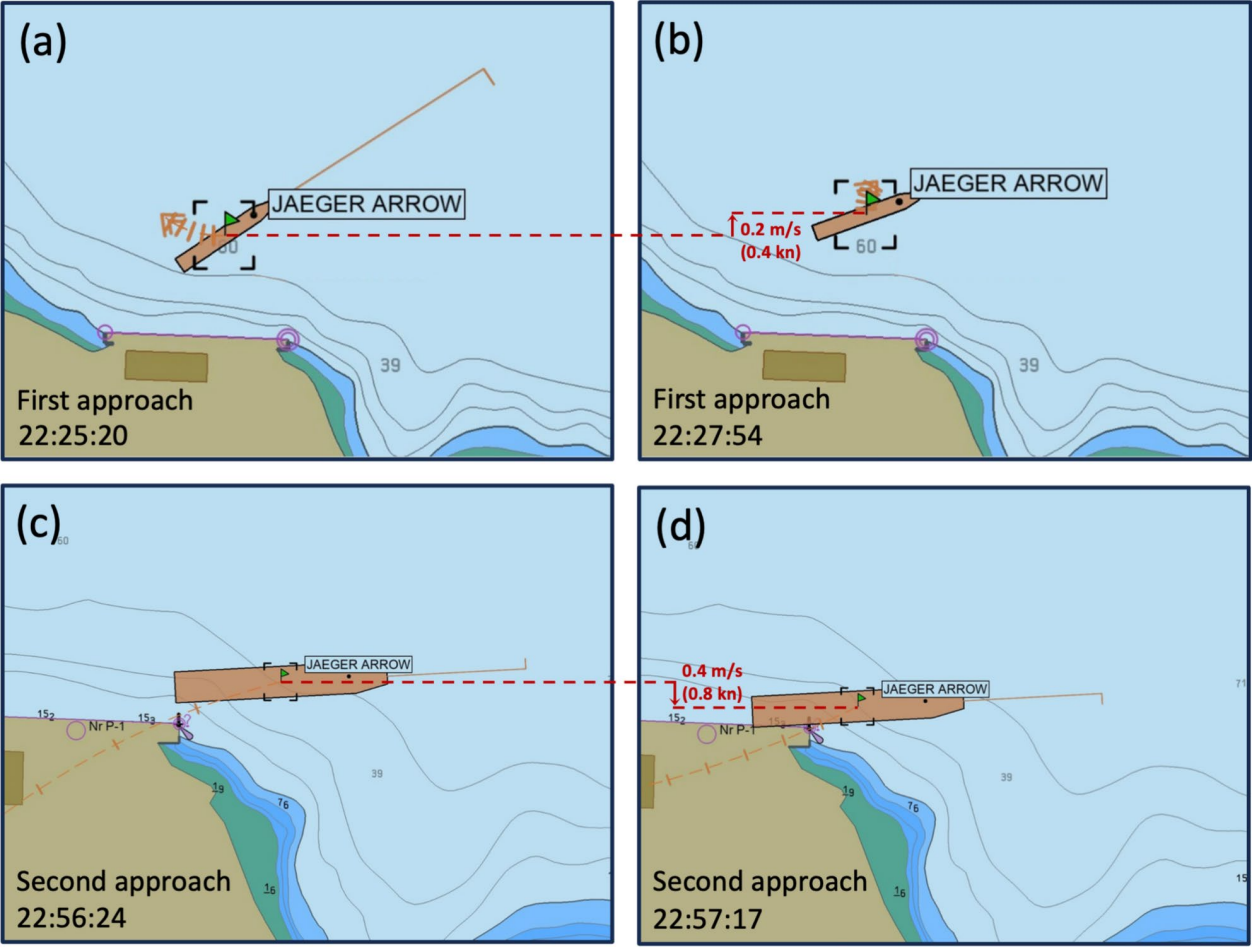
Uncontrolled transversal displacements of the MV *Jaeger Arrow* presumably caused by the presence of underwater waves incoming and reflecting off the wharf during the first (top panels) and second (bottom panels) docking approaches. During the first approach (**a**–**b**), the ship drifted northward over a distance of $\Delta x = 33~\textrm{m}$ during a period of $\Delta t = 154~\textrm{s}$, corresponding to an offshore drift speed $u_d = 0.2~\textrm{m}\,\textrm{s}^{-1}~(0.4~\textrm{kn})$ (red arrow in panel b). During the second approach (c-d), the ship drifted southward over a distance of $\Delta x = 21~\textrm{m}$ during a period of $\Delta t = 53~\textrm{s}$, corresponding to an inshore drift speed $u_d = 0.4~\textrm{m}\,\textrm{s}^{-1}~(0.8~\textrm{kn})$ (red arrow in panel d) before colliding with the wharf. The time is expressed in Coordinated United Time (UTC). The images are screenshots from the MV *Jaeger Arrow*’s navigation system.

On the evening of 30 September 2019, approximately an hour and a half after high tide during a spring tide and calm sea conditions, the Motor Vessel (MV) *Jaeger Arrow* (IMO 9215347, length 171 m, width 24.8 m, draught 10 m, Fig. 3, top panel) approached the Grande-Anse Terminal wharf in the Upper Saguenay Fjord (Fig. 4) for docking without the help of any tugboat. While carrying out her final backward approach maneuver, at around 22:25 UTC and a distance of about $150~\textrm{m}$ from the wharf (Fig. 5a), she was suddenly and unexpectedly displaced sideways offshore (i.e. northward) over a distance of $\Delta x = 33~\textrm{m}$ and over a period of $\Delta t = 154~\textrm{s}$, that is at a speed close to $0.2~\textrm{m}\,\textrm{s}^{-1}$ or $0.4~\textrm{kn}$ (Fig. 5a–b).

After this unsuccessful initial docking attempt, the vessel sailed away to reposition for a second approach; this time approaching the wharf much closer to avoid being deported offshore by the current of unknown origin that had compromised the first approach and that was presumably still there. Approximately 30 min later, just as the MV *Jaeger Arrow* was about to dock—about $10~\textrm{m}$ from the wharf — the vessel was unexpectedly displaced sideways at $0.4~\textrm{m}\,\textrm{s}^{-1}$ ($0.8~\textrm{kn}$). The vessel collided with the wharf at 22:57 UTC (Fig. 5c,d), causing damages to both the wharf and the ship, including a breach in the hull above the waterline.

**Fig. 6:**
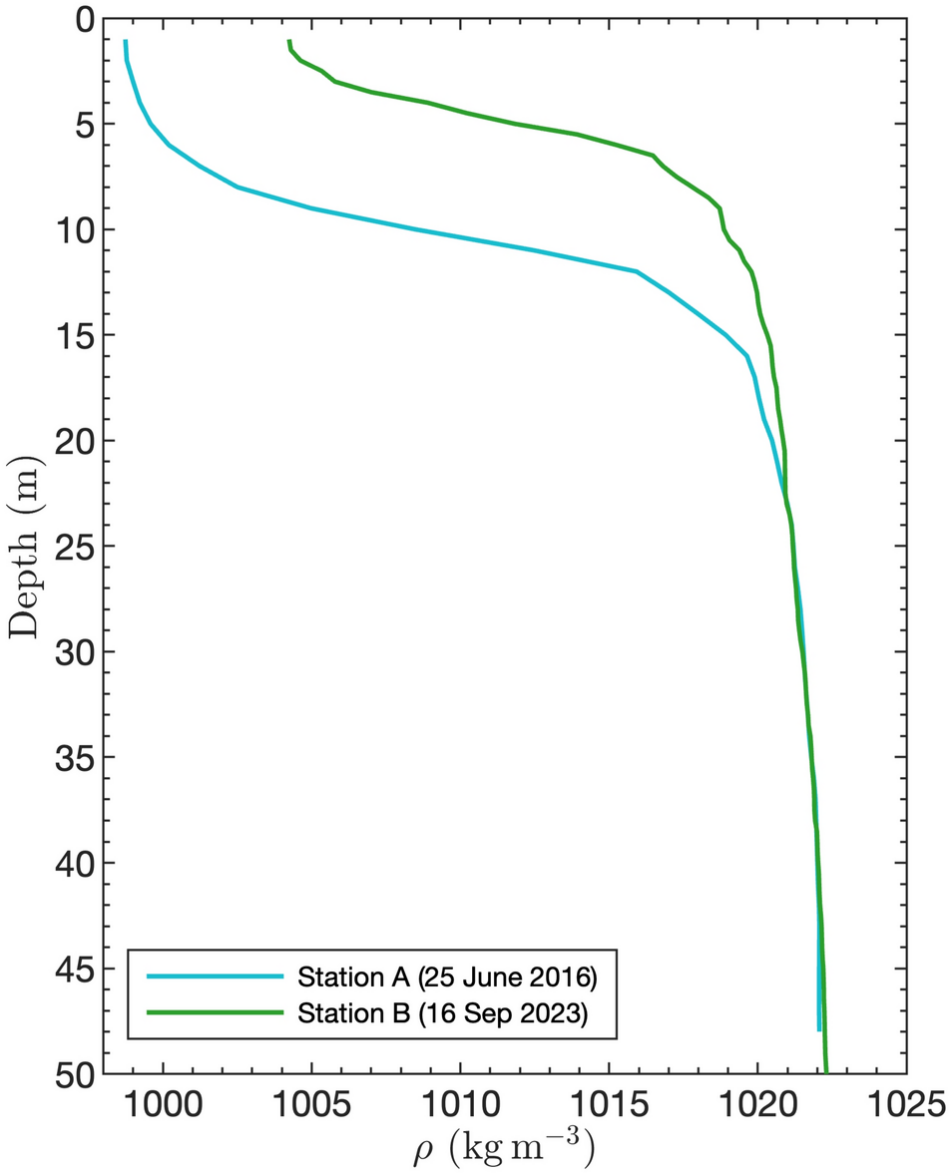
Density profiles observed off the wharf at stations A and B (see Fig. 4 for position) for two different years and seasons.

Determining the cause of the incident is of prime importance to prevent any such future event. Although specific details, such as the ship’s engine revolutions, rudder position, and other control data during the incident are unavailable, many circumstantial factors suggest that underwater waves may have been involved in the ship’s collision with the wharf based on the following:

- This area of the fjord is highly stratified (Fig. 6), with a 5 to 10-m thick brackish surface layer ($1000 \le \rho \le 1005~\textrm{kg}\,\textrm{m}^{-3}$) overlying a salty and dense bottom layer ($\rho \approx 1023~\textrm{kg}\,\textrm{m}^{-3}$), and subject to large tides of range up to about $5~\textrm{m}$. These are two elements favorable for the generation of large-amplitude underwater waves. Although not yet formally reported, it is very likely that this area of the fjord is subject to frequent occurrences of underwater waves as found in the other parts of the fjord (Fig. 1)^2,31,32^.
- The spatial and time scales that characterize the ship’s lateral displacements, i.e. $u_d = \Delta x/\Delta t \approx 0.2~\textrm{m}\,\textrm{s}^{-1}~(0.4~\textrm{kn})$ during the first approach and $u_d = \Delta x/\Delta t \approx 0.4~\textrm{m}\,\textrm{s}^{-1}~(0.8~\textrm{kn})$ during the second approach, is consistent with the passage of large-amplitude underwater waves of the type that can be regularly found in the Saguenay Fjord (Fig. 1)^2,8,31,32^.
- The fact that the ship was initially displaced offshore and then later on, inshore, is consistent with oscillatory motions that an incoming underwater wavetrain reflecting off the wharf and off the surrounding coastline could have caused; a process that has already been documented elsewhere in the Saguenay Fjord^31^. The timescale of $30~\textrm{min}$ is also consistent with the duration it would typically take an underwater wavetrain to approach and reflect off the coastline^31^.

It is within this context that we conducted this preliminary study to examine whether or not underwater waves could have caused the incident. To achieve this goal, we deployed a shore-based time-lapse camera as well as a series of temperature sensors along the wharf to see if underwater waves could be present in the vicinity of the Grande-Anse Terminal. We also examined with the help of theory and numerical simulations the properties and behaviour of underwater waves that could be expected in this area of the fjord. Finally, we interpret and discuss the results of these analyses in the context of ship manoeuvrability.

## Observations

### Naturally-occurring underwater waves

**Fig. 7:**
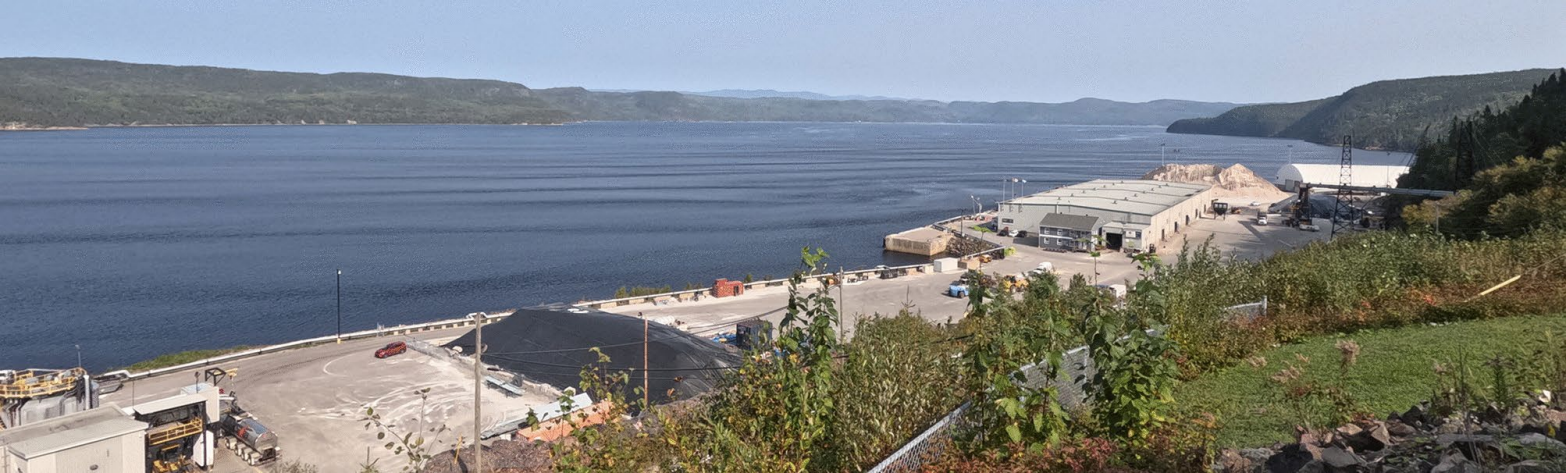
Field of view of the camera installed on the balcony of the Grande-Anse Terminal administration building and evidence of sea-surface signature of incident underwater waves seen as the banded pattern. This image is the same as the one georectified seen on panel (**b**) of Fig. 8 (27 September 2023 at 17:37:28 UTC).

**Fig. 8:**
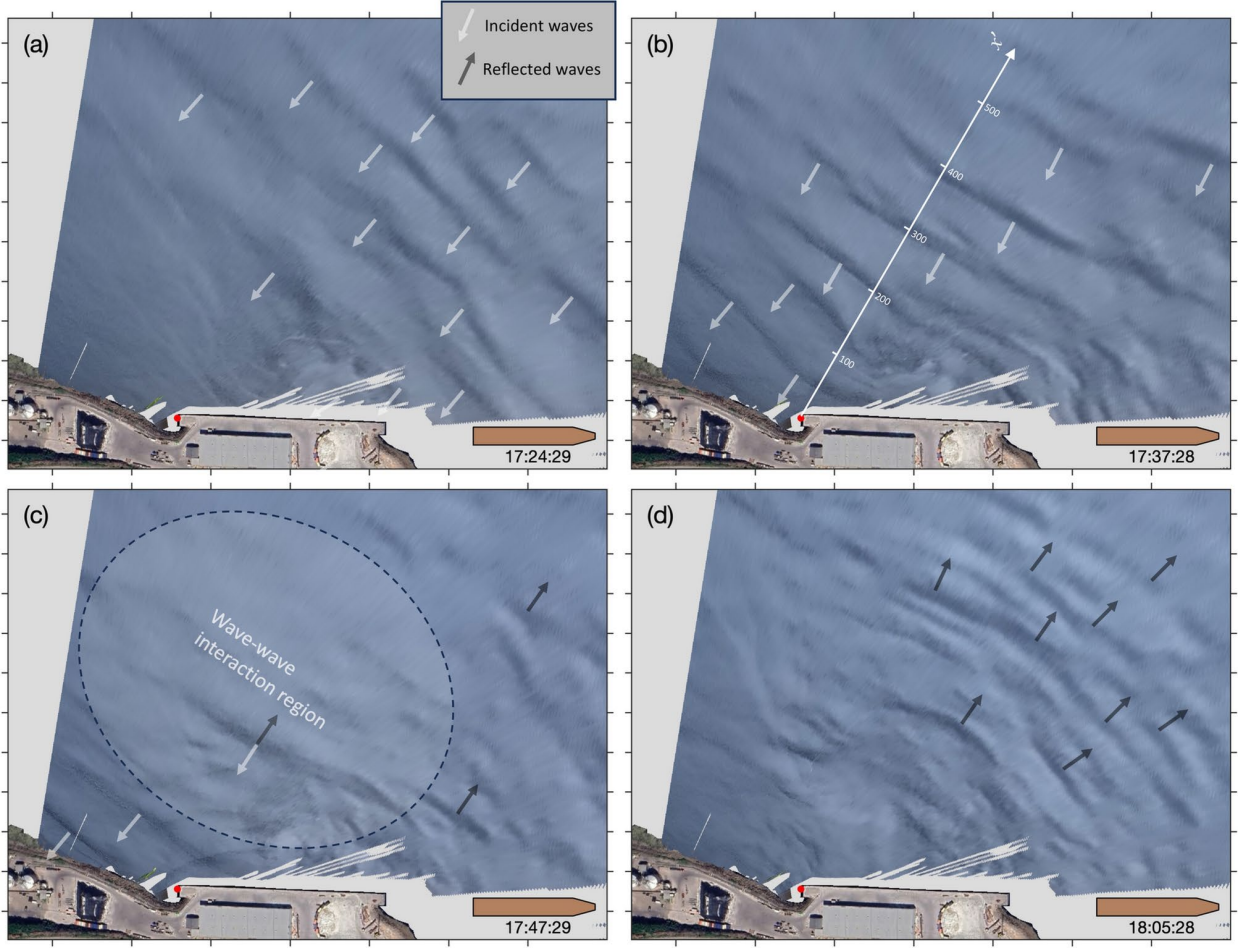
Sequence of georectified images, ordered chronologically as (**a**–**d**), showing the sea surface signature of naturally-occurring underwater waves interacting with the wharf. Photos taken on 27 September 2023. See Fig. 7 for the original field of view. For scale, the schematic ship depicted in the lower right corner of each panel has the size of the MV *Jaeger Arrow*, $171~\textrm{m} \times 25~\textrm{m}$. The red dot identifies the position of the thermistor chain. The direction of the underwater waves, illustrated by arrows, was determined from the sequence of consecutive images. The white axis in panel (**b**) is labeled in meters. A satellite image of the Grande-Anse terminal, identical to that in Fig 4, is overlaid on top of the georectified images.

A time-lapse camera was installed on the balcony of the Grande-Anse Terminal administration building for about three weeks in the fall of 2023 (see section Methods for details) in order to capture evidence of sea surface signatures of underwater waves as done previously elsewhere in the fjord (e.g. Fig. 1). As suspected, the camera captured several occurrences of underwater waves interacting with the Grande-Anse Terminal wharf. One of the clearest occurrence was recorded on 27 September 2023 around 17:30 UTC, approximately an hour before high tide, as seen by the alternation of smooth and rippled banded patterns, typical of those induced by underwater waves (Fig. 7). The georectified sequence of this event reveals an incident wavetrain, composed of about ten waves, approaching the wharf from the northeast (Fig. 8a-b). The distance between each incident wave is approximately 100 m, that is about four times the width of the MV *Jaeger Arrow*.

The wavetrain collides with the wharf and the shore (Fig. 8c) and reflects off mostly towards the northeast (Fig. 8d) but there is also some indications of wave reflection towards the northwest. The period during which the incident waves interact with the reflected waves, that is roughly around 17:47 (Fig. 8c), is characterized with blurry sea surface patterns that are more difficult to interpret, presumably because the currents associated with the reflected waves cancel out the currents associated with the incoming waves within this region. For this reason, we labeled this region and time period the ‘wave-wave interaction region’ (Fig. 8c). It is only once the incident wavetrain has completely passed that the reflected progressive waves really become detectable and trackable (Fig. 8d).

**Fig. 9:**
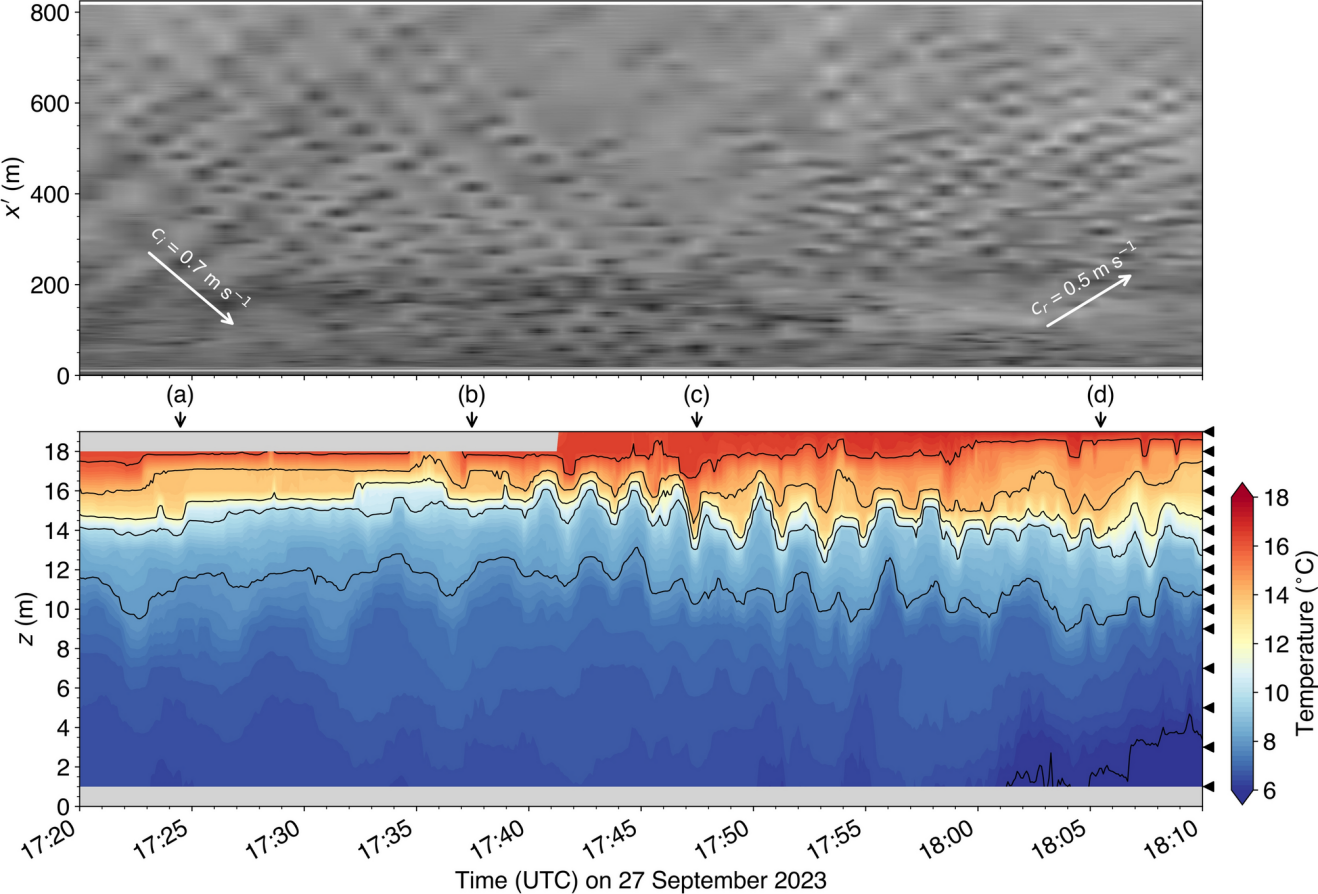
(top panel) Hovmöller diagram of sea-surface patterns along the $x'$ axis that points northeastward with its origin at the west corner of the wharf where the thermometers were deployed (see Fig. 8b) and, (bottom panel) observed temperature field at the wharf. The oblique wave rays in the space-time representation in the top panel appear discontinuous because the camera sampling interval ($60~\textrm{s}$) was too long to fully resolve the wave period. The white arrows represent the phase speed of the incident and reflected waves. The temperature data in the bottom panel were filtered with a 5-s moving average window before being decimated every $5~\textrm{s}$. The vertical arrows labeled from (**a**) to (**d**) indicate the times displayed in Fig. 8. The black triangles on the right indicates the height of the thermometers relative to the bottom.

For this exploratory field experiment, we also deployed a vertical chain of 15 thermometers at the western corner of the wharf (red dot on Fig. 8, see Methods for details). Coincident vertical oscillations in the temperature field recorded by the sensors confirm that mode-one underwater waves collided with the wharf during this sequence, starting from approximately 17:40 UTC (Fig. 9 bottom panel). The wave heights vary from 1 to $3~\textrm{m}$ and their period is about $2~\textrm{min}$.

The pixels intensity is also extracted from the sequence of images along the $x^\prime$ axis in Fig. 8b that goes from the temperature sensors toward the northeast, orthogonal to the waves. The resulting space-time Hovmöller diagram (Fig. 9 top panel) shows a series of V patterns that represent the wave reflection event. The first incident wave colliding with the wharf occurs around 17:39 (UTC), which corresponds approximately to the beginning of the oscillations observed in the temperature field at the thermocline depth. The incident wavetrain is composed of about ten waves, consistent with our estimation from the banded patterns observed in the images. The slopes of the incident oblique lines in the Hovmöller diagram represent the incident wave phase speed $c_i$. The incident slopes are remarkably constant which indicates that the phase speed of the incoming waves is constant as they approach the wharf. A linear best fit of these lines, which all have approximately the same slope, gives $c_i = 0.7~\textrm{m}\,\textrm{s}^{-1}$. The reflected waves are less well defined and show some curvature indicating a reduction in their phase speed as they propagated away. Their phase speed is smaller than their corresponding incident waves, presumably because some wave energy was lost during the interaction with the wharf and the bottom topography, with $c_r \sim 0.5~\textrm{m}\,\textrm{s}^{-1}$.

These observations confirm the presence of underwater waves in the vicinity of the Grande-Anse Terminal, and that these waves can collide with and reflect off the wharf.

### Ship-induced underwater waves

**Fig. 10:**
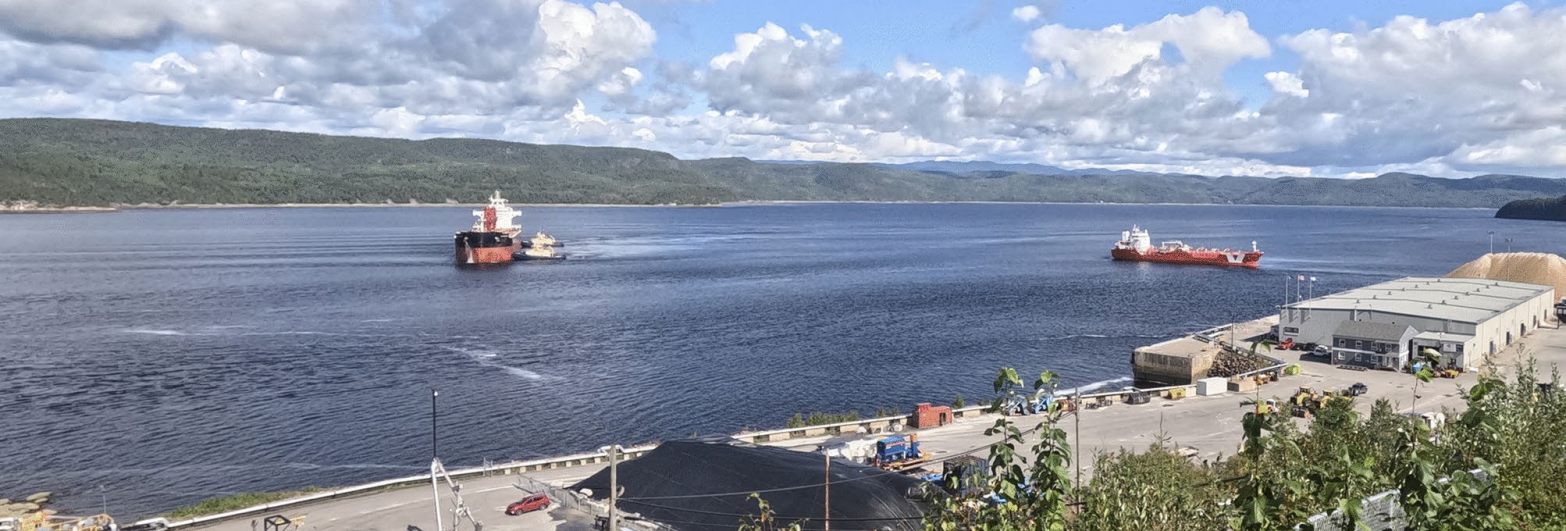
Maneuver of two ships, the MV *Santander* (left) and the MV *Iver Ambition* (right), around the Grande-Anse Terminal at 17:10 UTC on 20 September 2023. The banded sea-surface patterns are manifestation of underwater waves. These were possibly generated by the MV *Santander*. The MV *Santander* is $200~\textrm{m}$ long, $32~\textrm{m}$ wide, and has a draught of $7~\textrm{m}$.

**Fig. 11:**
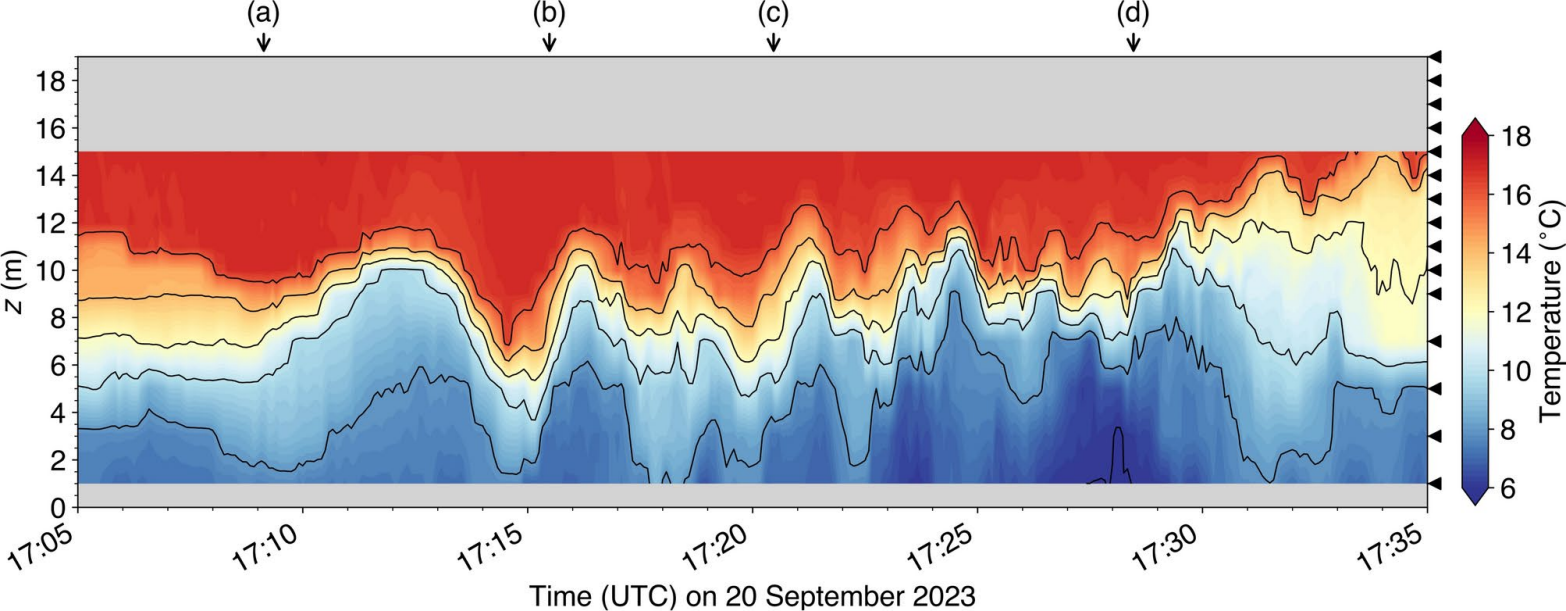
Similar to Fig. 9 bottom panel, but for the times displayed in Fig. 12.

As mentioned in the introduction, underwater waves can also be generated by passing ships. Among the images recorded by the camera, we found one occurrence of possibly ship-induced underwater waves (Fig. 10), coincident with some of the largest oscillations recorded by the temperature sensors, with height up to $6~\textrm{m}$ (Fig. 11).

On 20 September 2023, two ships were simultaneously manoeuvring around the Grande-Anse Terminal. The MV *Iver Ambition* (IMO 9439163) was leaving port while the MV *Santander* (IMO 9916305) was arriving, assisted by two tugboats (Fig. 10). The banded sea-surface patterns seen around the MV *Santander* indicates the presence of underwater waves. These waves have presumably been generated by the MV *Santander* herself while she was still further offshore and approaching the wharf. There are indeed some indications in the photos’ blurry far field (not shown) of such a coincidence, although this cannot be unambiguously confirmed. It is already known and documented that large ships such as the MV *Santander* can generate large-amplitude internal waves in the Saguenay Fjord^13^ and the possibility that a ship’s manoeuvrability may be affected by her own internal waves cannot be discarded.

**Fig. 12:**
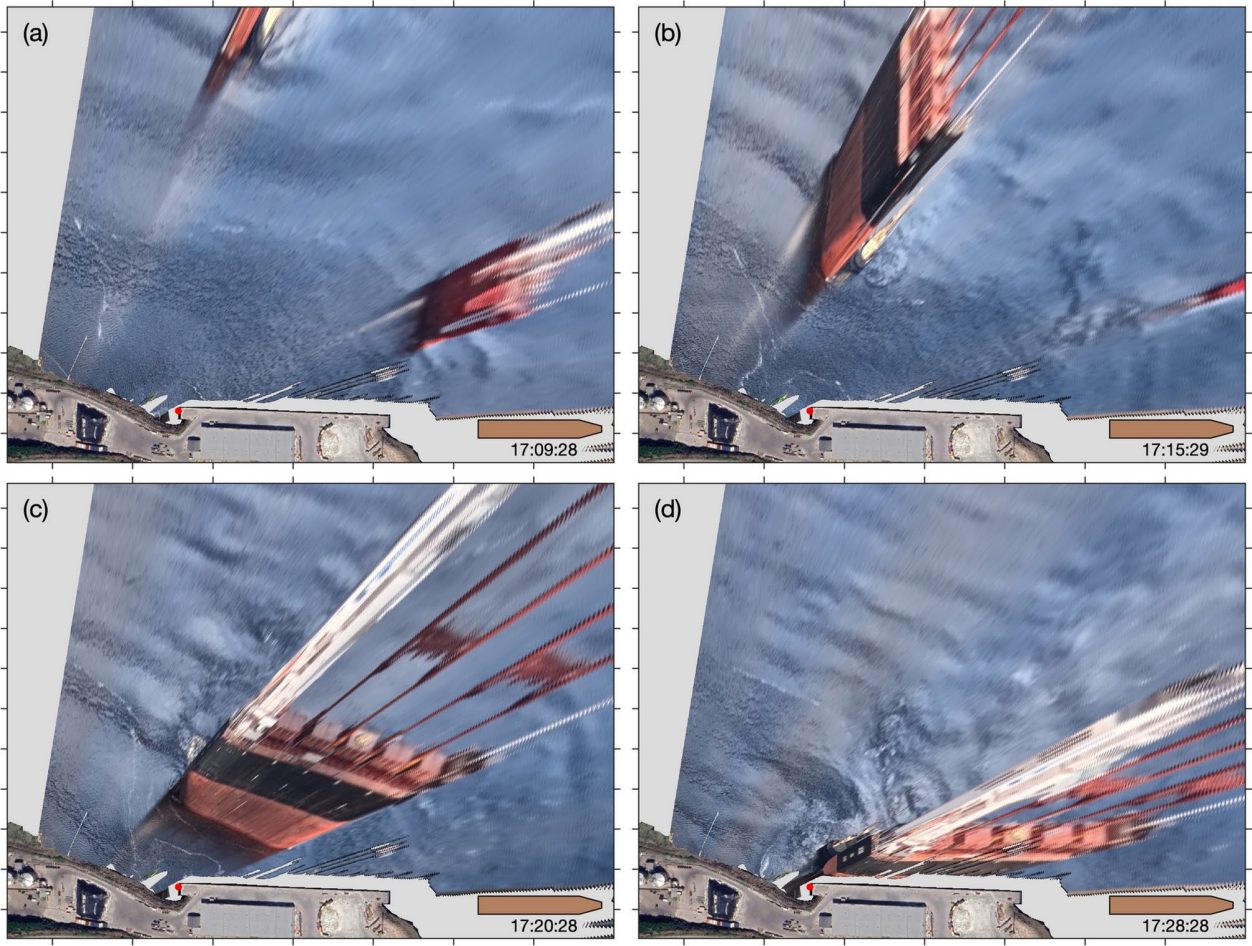
Similar to Fig. 8, but for the sequence of images, ordered chronologically as (**a**–**d**), showing the sea surface signature of, presumably, ship-induced underwater waves interacting with the wharf while the MV *Iver Ambition* was leaving port and the MV *Santander* was docking, assisted by two tugboats. Photos taken on 20 September 2023. Note that the *g_rect* algorithm^33^ used to georectify these images does not take into account elevation such that the ships appear projected onto the water, but everything at the level of the sea-surface is not distorted.

The georectified sequence of this event reveals that while the MV *Santander* approached the wharf, between 17:09:28 UTC and 17:15:29 UTC, at low tide, the incident wavetrain overtook her (Fig. 12a,b). The leading incident wave collided with the wharf during this period, and was the largest, as observed from the temperature field (Fig. 11). The following, and smaller, incident waves in the wavetrain collided with the wharf, and reflected offshore, during her final approach (Figs. 12b,c and 11). It appears that at the time of her docking, around 17:28:28 UTC, all the waves had reflected off toward the northeast (Figs. 12d and 11). One could expect that the docking of the MV *Santander* would have been compromised, considering the large underwater waves measured at the wharf. However, there is no indication from the images that there were any difficulties encountered during her docking, perhaps because she was assisted by two tugboats.

## Theory and simulations

### Theory

Ultimately, it is the wave-induced currents that is really required to determine if ship manoeuvrability can be compromised by the type of underwater waves observed here, but those are lacking in our measurements. In order to fill this gap, we infer here the wave-induced currents based on the steady-state, inviscid, fully nonlinear Dubreil-Jacotin-Long (DJL) theory (see Methods). Given the observed ambient density stratification and a prescribed wave available potential energy (i.e. a quantity proportional to wave amplitude squared), this theory can provide the corresponding wave shape, phase speed and, importantly for this study, the wave-current field. This idealized theory has proven to provide acceptable results of the structure and currents of internal solitary waves when compared to direct observations collected elsewhere in the Saguenay Fjord^31^.

Eight solutions using the two density profiles of Fig. 6 were obtained for different values of available potential energy (APE). The resulting wave properties are listed in Table 1, where *A* is the wave amplitude, $\lambda$ the half-width, *c* the phase speed, $\tau$ the half-period and $U_{max}$ the maximum horizontal current. These results show that increasing wave amplitude leads to stronger wave-induced currents and faster wave phase speed. The wavelength decreases with increasing wave amplitude, up to an upper limit (Table 1, Station B, $APE = 5\times 10^5~\textrm{kg}\,\textrm{m}\,\textrm{s}^{-2}$), consistent with the conjugate flow concept^34^ which predicts that the wavelength becomes longer as the amplitude reaches its limiting value. The wave-induced currents go from $\sim 0.10~\textrm{m}\,\textrm{s}^{-1}$ ($0.2~\textrm{kn}$) for the smallest wave to $0.95~\textrm{m}\,\textrm{s}^{-1}$ ($1.9~\textrm{kn}$) for the largest. The wave period, $\tau$, varies between 2 to $6~\textrm{min}$.

**Fig. 13:**
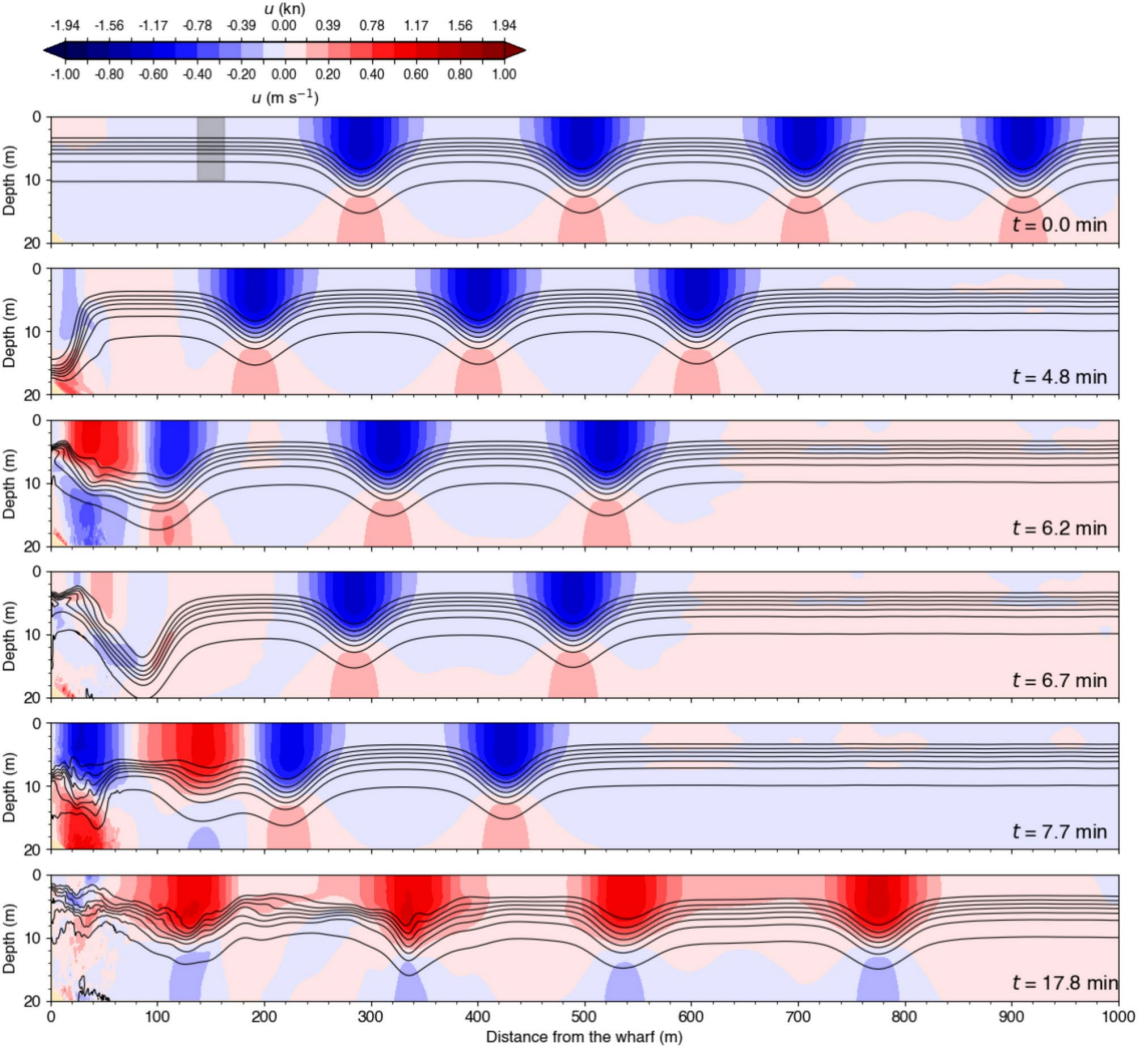
Results of an idealized simulation showing the impact of an underwater wavetrain against the Grande-Anse Terminal wharf. The wavetrain is composed of four identical medium-amplitude waves initialized with the DJL solver using the density profile from Station B with $APE = 1\times 10^5~\textrm{kg}\,\textrm{m}\,\textrm{s}^{-2}$. Density is shown in black contours with intervals of $2~\textrm{kg}\,\textrm{m}^{-3}$. The grey shaded rectangle in the top panel centered at $150~\textrm{m}$ from the wharf represents the width and draught of the MV *Jaeger Arrow*.

**Fig. 14:**
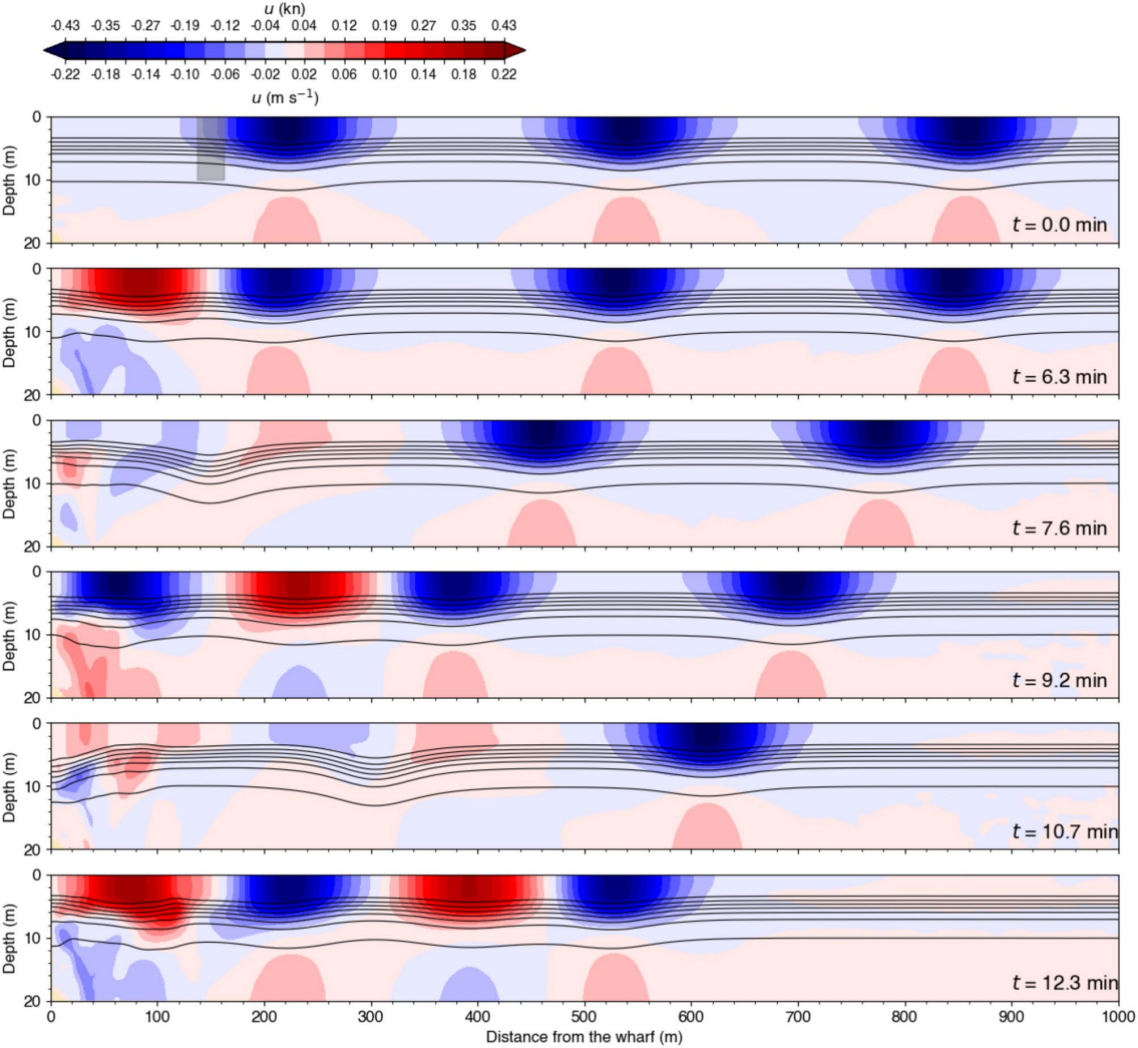
Similar to Fig. 13 except that the wavetrain is composed of four identical small-amplitude waves initialized using the density profile from Station B with $APE = 1\times 10^4~\textrm{kg}\,\textrm{m}\,\textrm{s}^{-2}$. Due to the longer wavelength, the fourth wave in the top panel is not visible, as it is located beyond $1000~\textrm{m}$. The colorbar differs from Figs. 13 and 15, having been adjusted to account for the smaller wave-induced currents.

**Fig. 15:**
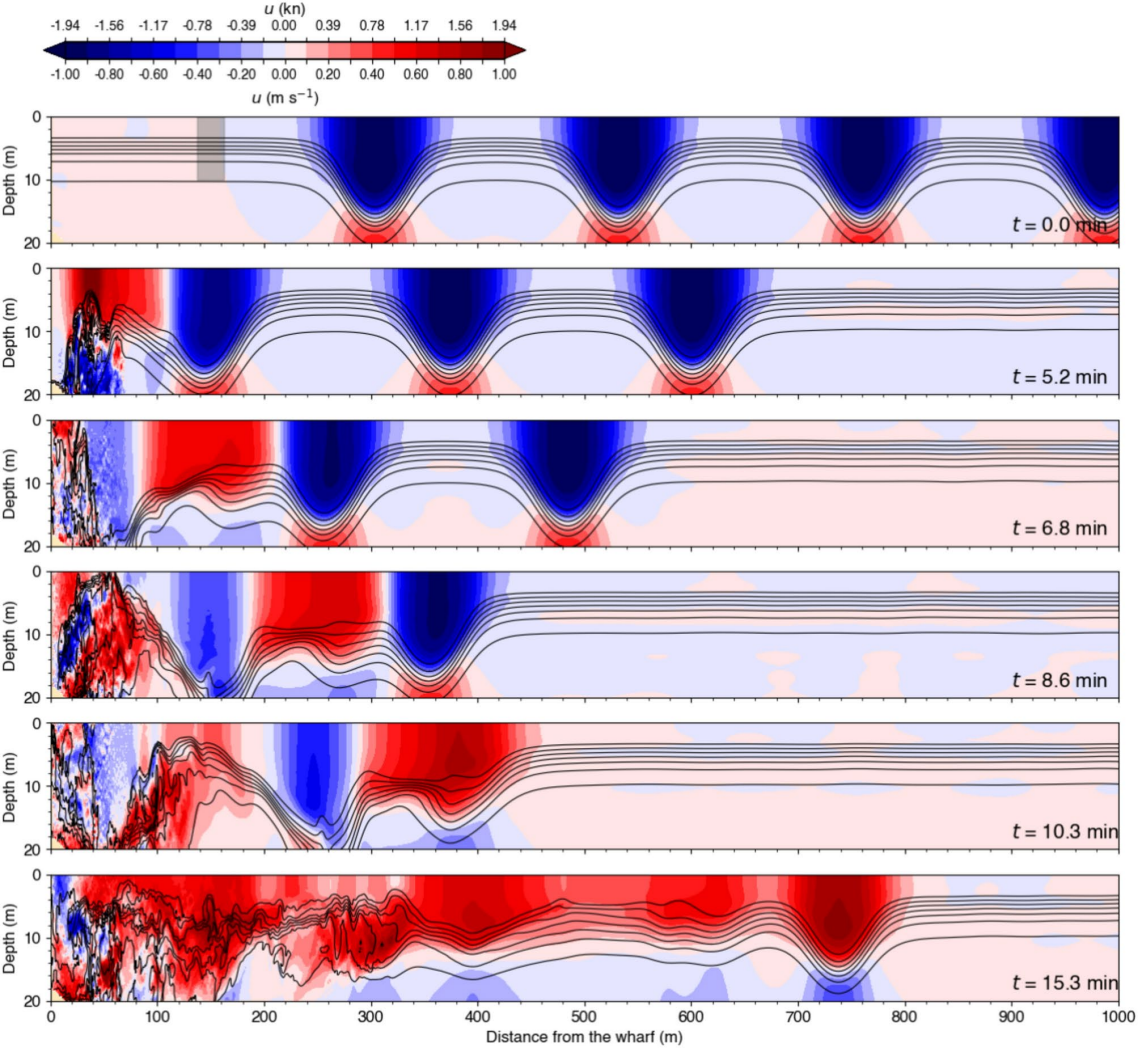
Similar to Fig. 13 except that the wavetrain is composed of four identical large-amplitude waves initialized using the density profile from Station B with $APE = 5\times 10^5~\textrm{kg}\,\textrm{m}\,\textrm{s}^{-2}$.

The top panel of Figs. 13, 14 and 15 show examples of the structure and the wave-induced currents for three of the eight theoretical waves calculated. For each case, the individual waves are repeated four times to produce wavetrains needed for the more realistic simulations discussed in the next section (see Methods). Indeed, internal solitary waves are rarely found completely alone but they are more generally found within wavetrains composed of several individual waves as seen in our observations presented above.

The density profile of 16 September 2023 (i.e. station B in Fig. 6) is the closest in time to our measurements and was used to calculate the theoretical properties of the underwater waves observed on the 27 September 2023. Given the observed wave heights, which ranged from 1 to $3~\textrm{m}$, the corresponding theoretical wave phase speed and period are approximately $0.9~\textrm{m}\,\textrm{s}^{-1}$ and $3~\textrm{min}$. These values differ slightly from the observed $0.7~\textrm{m}\,\textrm{s}^{-1}$ and $2~\textrm{min}$. This difference between theory and observations could be attributed to the fact that the DJL theory does not account for the potential influence of complex background currents interfering with the underwater waves; a difficult problem that could eventually be dealt with using more realistic numerical simulations.

**Table 1:** Properties of leftward-propagating theoretical underwater waves generated with the Dubreil-Jacotin-Long (DJL) equation solver using several density profiles and several values for the imposed available potential energy (APE) per unit width.

CTD profile	Station A				Station B			
$\textrm{APE}~(\textrm{kg}\,\textrm{m}\,\textrm{s}^{-2})$	$1 \times 10^4$	$5 \times 10^4$	$1 \times 10^5$	$5 \times 10^5$	$1 \times 10^4$	$5 \times 10^4$	$1 \times 10^5$	$5 \times 10^5$
$A~(\textrm{m})$	0.9	2.3	3.5	8.1	1.6	4.1	5.8	11.7
$\lambda ~(\textrm{m})$	453	297	259	225	210	145	137	151
$c~(\textrm{m}\,\textrm{s}^{-1})$	1.24	1.3	1.33	1.44	0.86	0.97	1.04	1.21
$\tau ~(\textrm{s})$	364	229	194	156	243	149	131	124
$U_{max}~(\textrm{m}\,\textrm{s}^{-1})$	0.10	0.26	0.37	0.74	0.13	0.39	0.58	0.95

### Simulations

While the DJL theory presented above can provide useful information about the waves steady-state structures and associated currents, it cannot provide information about the complex behaviour caused by wave reflection and breaking against the wharf. For this, we use the prognostic, nonhydrostatic and fully nonlinear MITgcm hydrodynamic numerical model^35^ set in a two-dimensional configuration with a realistic bathymetry that include the vertical wharf at its leftward boundary. The initial condition is a leftward-propagating wavetrain composed of four consecutive identical underwater waves issued from the DJL theory (see Methods for details). Three simulations were carried out with small, medium and large amplitude waves.

Figure 13 shows the density field and wave-induced horizontal currents at different stages for the simulation initialized with a medium amplitude wavetrain. At $t = 0~\textrm{min}$, the four waves propagate leftward towards the Grande-Anse wharf with the current above the pycnocline, that is roughly in the top $10~\textrm{m}$, being in the same direction as the wave propagation (blue), that is towards the wharf, while the current below the wave interface is directed in the opposite direction, offshore (red). This offshore-oriented deep current is typically below the ship draught and should not affect ship behaviour.

The first incident wave collides against the wharf at $t = 4.8~\textrm{min}$, and then reflects at $t = 6.2~\textrm{min}$ (red in the top layer near the wharf), just before interfering with the second incident wave at $t = 6.7~\textrm{min}$. At $t = 7.7~\textrm{min}$, the second incident wave collides with the wharf, while the first reflected wave is about to interfere with the third incident wave. At $t = 17.8~\textrm{min}$, all four waves have reflected and propagate away from the wharf. During a wave-wharf and a wave-wave interaction, such as seen at $t = 4.8~\textrm{min}$ at the wharf and at $t = 6.7~\textrm{min}$ about $85~\textrm{m}$ from the wharf in Fig. 13, the horizontal wave-induced currents nearly vanish momentarily, because in opposite directions at $t = 6.7~\textrm{min}$, while the downward displacement of the pycnocline is at its maximum. These weak horizontal currents during interactions support our interpretation of the ‘wave-wave interaction region’ depicted in Fig. 8c.

The small-amplitude underwater wave simulation is similar to the medium-amplitude underwater wave, but with weaker wave-induced currents and a longer wavelength (Fig. 14). The ‘wave-wave interaction region’ is visible when the first reflected wave interacts with the second and third incident waves: at $t = 7.6~\textrm{min}$ about $150~\textrm{m}$ from the wharf, and at $t = 10.7~\textrm{min}$ about $310~\textrm{m}$ from the wharf, respectively. At $t = 10.7~\textrm{min}$, the second incident wave also collides with the wharf. At this stage, only the horizontal currents associated with the fourth incident wave is momentarily significant.

In the large-amplitude underwater wave simulation (Fig. 15), the wave-induced currents are stronger, and the wavelength is longer compared to the medium-amplitude underwater wave, as the wave height has reached its theoretical limit, as mentioned in the previous section. The dynamics of the waves interacting with the wharf is much more complex, making it difficult to distinguish the currents associated with subsequent reflected waves after the first reflection due to the high turbulence generated by the large-amplitude wavetrain.

**Fig. 16:**
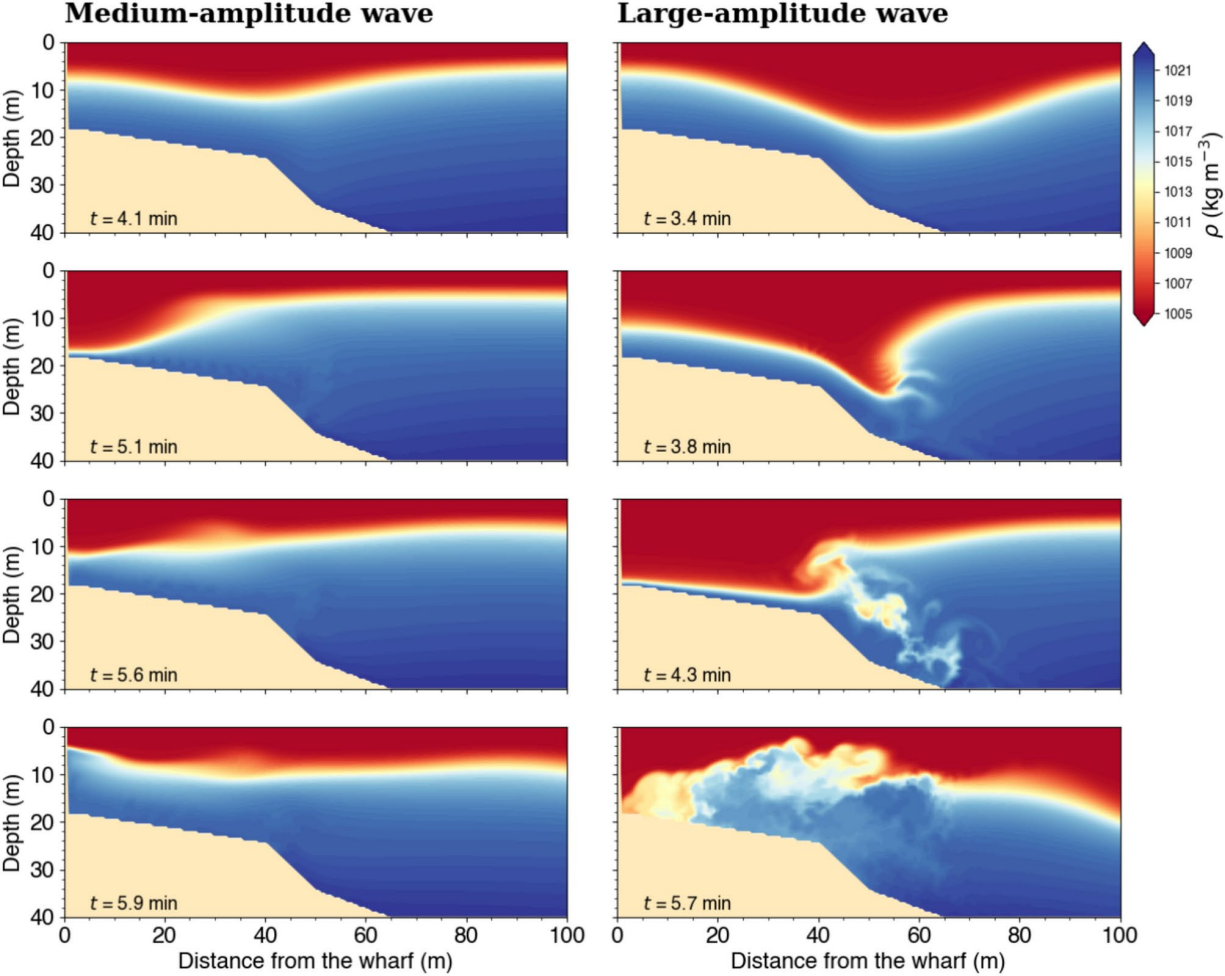
Density profiles for the medium- (left column) and large-amplitude (right column) simulated underwater wavetrains from Figs. 13 and 15, respectively.

As the large-amplitude wave shoals as it approaches the wharf (Fig. 16, right column), it steepens ($t = 3.8~\textrm{min}$) and eventually breaks ($t = 4.3~\textrm{min}$) which generates turbulence above the seafloor ($t = 5.7~\textrm{min}$). The wave breaking is due to the very large height of the wave interacting with the topography. In contrast, the medium-amplitude underwater wave (Fig. 16, left column) does not reach breaking condition, even if the wave height increases when it collides with the wall, as it does not reach the topography before its collision.

**Fig. 17:**
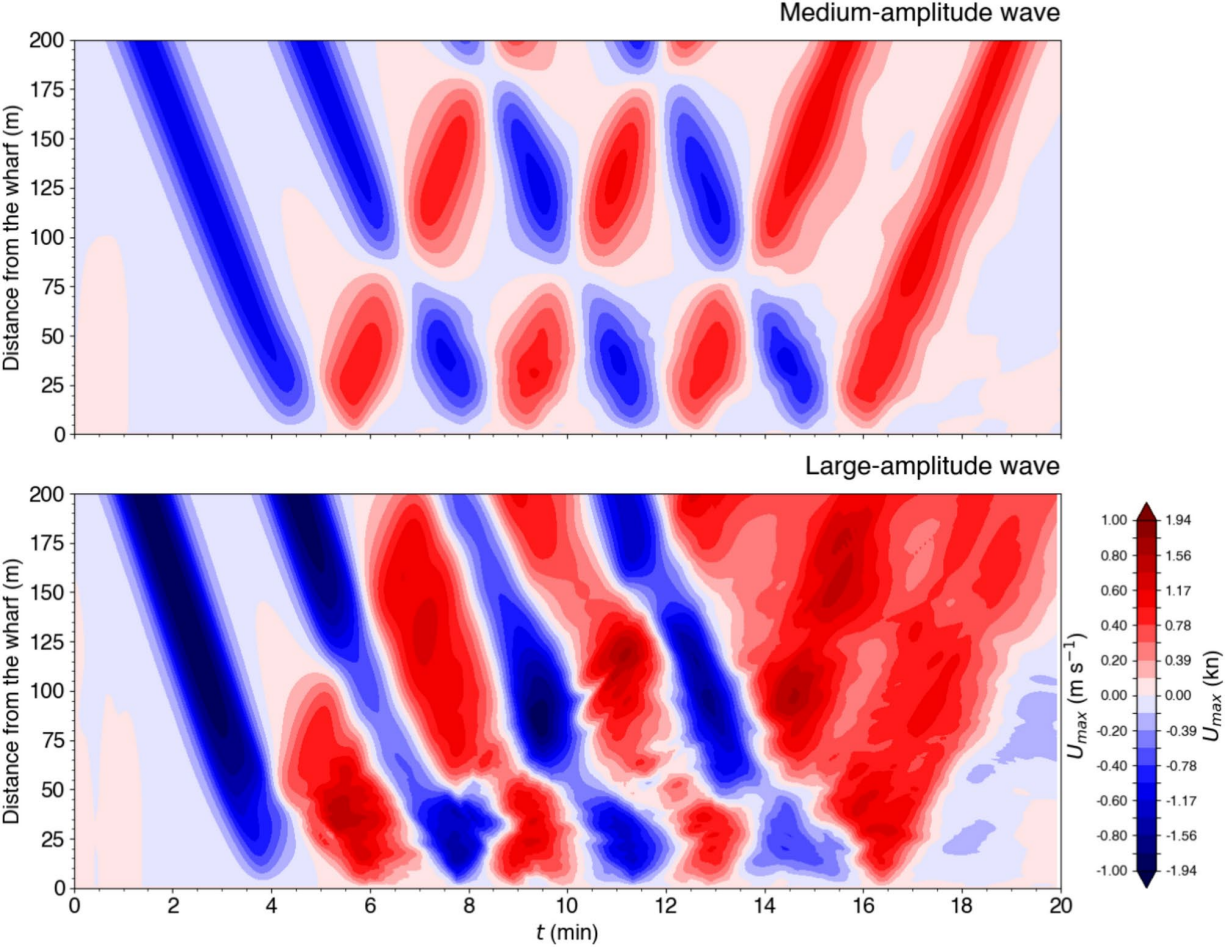
Hovmöller diagram of the depth-averaged velocity over $10~\textrm{m}$ for the medium- (top panel) and large-amplitude (bottom panel) simulated underwater wavetrains from Figs. 13 and 15, respectively.

A Hovmöller diagram, similar to the one constructed from the time-lapse photography (Fig. 9), of the wave-induced currents illustrates their evolution in space and time (Fig. 17). In this representation, the blue and red slanted beams represent incident and reflected wave-induced currents, respectively. The first medium-amplitude incident wave (Fig. 17, top panel) propagates toward the wharf between 0 and $4~\textrm{min}$ (blue), collides with the wharf at around $5~\textrm{min}$ (near-zero currents), and then reflects off the wharf between 6 and $10~\textrm{min}$ (red). The discontinuity between the incident (blue) and reflected (red) beams marks the ‘wave-wave interaction region’ where the horizontal currents are very weak, and occurs at two locations relative to the wharf: around $80-90~\textrm{m}$ for the first reflected – second incident (at $\sim 7~\textrm{min}$), second reflected – third incident (at $\sim 10~\textrm{min}$) and third reflected – fourth incident (at $\sim 14~\textrm{min}$) interactions; and around $180-190~\textrm{m}$ for the first reflected – third incident (at $\sim 8.5~\textrm{min}$) and second reflected – fourth incident (at $\sim 12~\textrm{min}$) interactions. The fourth reflected wave propagates away from the wharf between 16 and $20~\textrm{min}$. The Hovmöller diagram for the small-amplitude wave simulation is not shown here, as it closely resembles the medium-amplitude case (Fig. 17, top panel), with differences in the beam slope and the locations of the ‘wave-wave interaction region’.

The Hovmöller diagram for the large-amplitude wavetrain simulation (Fig. 17, bottom panel) differs remarkably from the smaller-amplitude wavetrain presented above and is more difficult to interpret due to the complex nature of wave breaking. One notable difference is that the ‘wave-wave interaction region’ that appears in the smaller amplitude cases, where the horizontal currents are very weak at the intersection of the blue and red beams, is not clearly present here. Other processes are at play given the complexity of the turbulent flow.

Another remarkable difference is that the red beams, i.e. those of positive slopes that were associated with the reflecting waves in the previous case, appear here to slant in the same direction as the blue beam, that is as the incident waves. In reality, this is a false impression as two processes are superimposed here. A good portion of the incident waves are reflected as red beams of positive slopes, as in the previous case. These are harder to see here but can be guessed by visually connecting the locations of maximum red intensity. These hardly discernible red beams of reflected waves are superimposed on predominant offshore surface currents associated with the shoreward advancing of the internal swash and intrusion caused by wave breaking (Fig. 16, right panel); a process that can be seen, for example, in the numerical simulations of Vlasenko and Hutter^36^ on the breaking of internal solitary waves over slopes (see their Fig. 7). Note that there is a sign error in the horizontal velocity reported in Figs. 3 and 7 of Vlasenko and Hutter^36^ (Vasiliy Vlasenko, personal communication). For consistency with the orientation of the distance axis, the negative velocity should be positive, and vice versa.

Finally, the large zone of rightward currents, spanning from $16~\textrm{min}$ across the entire $200~\textrm{m}$, indicates that the reflected waves are not dissociated from each other compared to the incident wavetrain but have somewhat merge together during the reflection and breaking processes to produce a longer, less wavy current structure.

## Discussion

The images and thermometers deployed at the Grande-Anse Terminal during the fall of 2023 captured several occurrences of underwater waves colliding with and reflecting off the wharf. The clearest event (Figs. 7 and 8) has been analysed in greater detail and revealed a wavetrain composed of about a dozen waves with phase speed of $0.7~\textrm{m}\,\textrm{s}^{-1}$, heights ranging from 1 to $3~\textrm{m}$, and periods of approximately $2~\textrm{min}$ (Fig. 9). Relative to the local tide, this event occurred approximately one hour before high water. In comparison, the incident of the MV *Jaeger Arrow* occurred about one and a half hours after local high water. Horizontal wave-induced currents, estimated using the DJL theory, range from 0.1 to $0.3~\textrm{m}\,\textrm{s}^{-1}$, equivalent to 0.2 to $0.6~\textrm{kn}$, and should be confirmed through observations in future fieldwork. Typically, transverse currents exceeding 0.3 to $0.5~\textrm{kn}$, particularly when they arise unexpectedly within a minute or two, could well compromise docking approach maneuvers.

Our results offer a plausible explanation for the particular behaviour that the MV *Jaeger Arrow* experienced as she approached the wharf that evening of September 2019, being initially deported offshore and later on inshore towards the wharf against which she collided. The ship may have been caught within an underwater wavetrain, with a reflected wave initially displacing her offshore, followed by a shoreward push toward the wharf from a subsequent incoming wave.

Our analysis suggests that docking a large ship such as the MV *Jaeger Arrow* in the presence of reflecting underwater waves could be challenging. As a ship goes through a reflecting wavetrain, it encounters regions influenced both by incident and reflected waves as well as unpredictable moments when the interaction of the incident and reflected waves is such that the wave-induced current is almost nil, giving a false sense of momentarily calm conditions, as illustrated in Figs. 13–15 and 17. Additionally, docking conditions would depend on whether the underwater waves break. A wavetrain of breaking waves would produce significantly more turbulence, creating a more complex and less predictable environment, as seen in Fig. 16.

A second event of interest shows the presence of an underwater wavetrain colliding with and reflecting off the wharf while the MV *Santander* ($200~\textrm{m}$ long, $32~\textrm{m}$ wide) was docking with the assistance of two tugboats (Figs. 10 and 12). The vertical displacements of the isotherms measured at the wharf during this event reached up to $6~\textrm{m}$ (Fig. 11). The interpretation of the images suggests that these waves were generated by the MV *Santander* herself, in a manner similar to how other large vessels have been documented to generate underwater waves while steaming in the Saguenay Fjord^13^. Generation likely occurred while the MV *Santander* was still steaming further offshore before being assisted by the tugboats. Additionally, evidence of underwater waves generated by the MV *Jaeger Arrow* herself as she navigated the Saguenay Fjord has, coincidentally, been captured during a previous fieldwork (Fig. 3, middle and top panels). Based on these observations, it cannot be entirely ruled out that the approach of the MV *Jaeger Arrow* was similarly compromised by her own underwater waves. This is an important and unexpected result of this research that should be further investigated.

Since this study is limited to two-dimensional simulations, we focused on the idealized scenario where underwater waves collide with and reflect off perpendicularly to the Grande-Anse wharf. However, as shown in Figs. 8 and 12, the angle of wave incidence is not necessarily perpendicular. Future research should involve three-dimensional simulations of underwater waves interacting with the wharf at varying angles of incidence to better replicate realistic conditions. Additionally, our simulations assume no background currents. In reality, complex background currents, such as shear layer currents^37^, likely influence the behavior and properties of underwater waves and should be considered in future studies.

Ultimately, integrating three-dimensional fields of wave-induced currents into pilot simulators could greatly improve training programs for ship pilots, allowing them to navigate more safely in the presence of these waves and their associated currents. Moreover, collecting more data of underwater waves in the vicinity of the Grande-Anse terminal in order to provide some statistics about their occurrence relative to tidal conditions would be highly valuable to guide pilots and harbour masters. Such data could help raise awareness and optimize berthing procedures during periods when underwater waves are likely to be prevalent, thereby improving both safety and operational efficiency at the terminal. Lastly, if there is a risk that cargo ships might be compromised by their own underwater waves during docking, some procedures and guidelines could be implemented to mitigate this risk and ensure secure docking operations.

## Methods

### Observations

The density profiles in Fig. 6 were collected at two stations (Fig. 4) in the Saguenay Fjord with a Sea-Bird SBE9 Conductivity Temperature Depth (CTD) profiler. Station A ($48^{\circ }$ $25.40^\prime$ N, $70^{\circ }$ $51.94^\prime$ W) was visited by the Atlantic Zone Monitoring Program (AZMP) from Fisheries and Oceans Canada (DFO), and Station B ($48^{\circ }$ $24.81^\prime$ N, $70^{\circ }$ $47.53^\prime$ W) was monitored by the Institut des Sciences de la Mer (ISMER) *R/V Coriolis II* Survey.

In order to measure the temperature variations caused by incoming underwater waves (Fig. 9), we deployed at the western corner of the Grande-Anse Terminal wharf ($48^{\circ }$ $24.107^\prime$ N, $70^{\circ }$ $50.001^\prime$ W, red dots in Fig. 8) a vertical chain composed of 14 RBR${ solo}^3$ T temperature sensors and one RBR${ duet}^3$ T.D temperature-depth sensor. The deepest instrument was the temperature-depth sensor and was positioned at one meter above the bottom. The following 4 sensors were positioned 2 meters apart and the remaining were set 1 meter apart (left-pointing triangles in the bottom panel of Fig. 9). The sensors covered a total depth of 19 m and, considering that the water depth at the wharf is 13.8 m at mean low water, around 5 sensors were out of the water during low tides. The sensors recorded data every second from 1315 (UTC) 15 September 2023 until 1645 (UTC) 1 November 2023.

Simultaneously, a CamDo Time Lapse GoPro HERO11 Camera of resolution 5568 $\times$ 4872 pixels was installed on the balcony of Port Saguenay authority offices ($48^{\circ }$ $24.050^\prime$ N, $70^{\circ }$ $50.180^\prime$ W) to record the sea surface signature of underwater waves (Fig. 7). Images were taken every minute during daytime (1000 to 2300 (UTC)) from 1840 (UTC) 15 September 2023 until 1745 (UTC) 5 October 2023. The oblique images were then georectified using the *g_rect* Matlab toolbox^33,38,39^ (Fig. 8). This technique has proven to provide an invaluable mean to detect the presence of underwater waves in the Saguenay Fjord^8,31^ and the St. Lawrence Estuary^17^.

### Theory

Underwater waves characteristics can be described by the fully nonlinear, steady-state, two-dimensional Dubreil-Jacotin-Long (DJL) theory^31,37^. Assuming no background current and a flat bottom, the DJL equation for the isopycnal displacement, $\eta (x, z)$, subject to the boundary conditions $\eta ~=~0$ at the surface and bottom, and $\eta ~{\rightarrow }~0$ as $x~{\rightarrow \pm \infty }$, can be written as:

1 $$\begin{aligned} \nabla ^2 \eta + \frac{N^2(z-\eta )}{c^2} \eta = 0, \end{aligned}$$

where *c* is the wave phase speed, and *N*(*z*) is the background Brunt-Väisälä buoyancy frequency, defined as:

2 $$\begin{aligned} N(z) = \sqrt{- \frac{g}{\rho _0} \rho _z}, \end{aligned}$$

where $g~=~9.81~\textrm{m}\,\textrm{s}^{-2}$ is the gravitational acceleration, $\rho _z$ is the partial derivative with respect to the vertical position, *z*, of the background density, $\rho$, and $\rho _0$ is the reference density defined as the maximum of $\rho$ in this study. Considering the density profiles collected near the Grande-Anse terminal in Fig. 6 and an imposed available potential energy, with values of $1\times 10^4$, $5\times 10^4$, $1\times 10^5$ or $5\times 10^5~\textrm{kg}\,\textrm{m}\,\textrm{s}^{-2}$, we inferred mode-one solutions to equation 1 using the DJL Equation Solver (DJLES) Python package^40^. The DJLES domain is $800~\textrm{m}$ long and $50~\textrm{m}$ deep with horizontal and vertical resolutions, $\Delta x = 2~\textrm{m}$ and $\Delta z = 0.5~\textrm{m}$ respectively. Once $\eta$ and *c* are obtained, the wave-induced density and velocity fields can be determined from the streamfunction of the wave-induced motion.

### Numerical model

2D numerical simulations of underwater waves colliding with the Grande-Anse Terminal wharf are carried out with the Boussinesq, fully nonlinear, free-surface and nonhydrostatic MITgcm hydrodynamic ocean model^35^. The simulations are performed in the vertical $x-z$ plane. The domain is $2~\textrm{km}$ long with an idealized bathymetry of Saguenay Fjord taken perpendicular to the Grande-Anse Terminal wharf, as illustrated in Fig. 16. The depth at the wharf corresponds to the total depth during the incident. On the evening of 30 September 2019, the predicted tidal water level, relative to chart datum, during the time of the approach and docking of the MV *Jaeger Arrow* was around $4.5~\textrm{m}$. Given that the depth at the wharf at mean low water is $13.8~\textrm{m}$, the depth in our simulations is chosen to be $18.3~\textrm{m}$. The horizontal resolution is $\Delta x = 0.5~\textrm{m}$ over the first $300~\textrm{m}$ near the wharf, and increases by 10$\%$ until reaching $2~\textrm{m}$. The vertical resolution is constant with $\Delta z = 0.5~\textrm{m}$.

An underwater wavetrain, composed of four identical waves, is initially positioned in the flat bottom section of the domain (Figs. 13,  14 and 15, top panel). The waves are generated by solving the DJL equation, as described in the previous section. They are equally spaced from each other by half their wavelength. Three simulations are initialized with the wave-induced density and velocity fields from the DJL solution. This choice allows us to study the impact of different wave amplitudes on the wharf using the background density profile the closest in time to the incident, that is Station B on 16 September 2023 (Fig. 6).

Momentum is advected with a second-order centered scheme with constant and equal horizontal and vertical viscosities $\nu _h = \nu _v = 5 \times 10^{-4}~\textrm{m}^{2}\,\textrm{s}^{-1}$. Since the model must be initialized with temperature and salinity fields and that DJL provides us with the density field only, we use, for simplicity, a linear equation of state with thermal and haline expansion coefficients, $\alpha = 0$ and $\beta = 7.5\times 10^{-4}~\mathrm {(g\,kg^{-1})^{-1}}$ respectively, to produce the initial temperature and salinity fields. Advection of temperature and salinity is achieved with the Monotonized Central flux limiter scheme^41^. The horizontal and vertical eddy diffusivities are set to zero, i.e. $\kappa _h = \kappa _v = 0$. A free-slip boundary condition is applied at the side-walls. At the bottom, the stress is parameterized with a quadratic drag following the logarithmic law of the wall with a bottom roughness length of $0.01~\textrm{m}$. The free-surface is treated implicitly and is thus unconditionally stable.

The duration of each simulation is $60~\textrm{min}$ such that all four underwater waves travel back to their origin after reflecting off the wharf. The time step $\Delta t = 0.1~\textrm{s}$ is chosen such that it respects the CFL conditions for flow speed and viscosity.

## Data Availability

All data used in this study are archived in the Dryad repository: https://doi.org/10.5061/dryad.ksn02v7fz.

## Code availability

The DJLES code used to produce the internal solitary waves is available here: https://github.com/mdunphy/DJLES. The MITgcm code is available here: https://github.com/MITgcm/MITgcm.
